# Mortality from tetanus between 1990 and 2015: findings from the global burden of disease study 2015

**DOI:** 10.1186/s12889-017-4111-4

**Published:** 2017-02-08

**Authors:** Hmwe H. Kyu, John Everett Mumford, Jeffrey D. Stanaway, Ryan M. Barber, Jamie R. Hancock, Theo Vos, Christopher J. L. Murray, Mohsen Naghavi

**Affiliations:** Institute for Health Metrics and Evaluation, University of Washington, 2301 5th Ave. Suite 600, Seattle, WA 98121 USA

**Keywords:** Tetanus, Mortality, Distribution, Trends

## Abstract

**Background:**

Although preventable, tetanus still claims tens of thousands of deaths each year. The patterns and distribution of mortality from tetanus have not been well characterized. We identified the global, regional, and national levels and trends of mortality from neonatal and non-neonatal tetanus based on the results from the Global Burden of Disease Study 2015.

**Methods:**

Data from vital registration, verbal autopsy studies and mortality surveillance data covering 12,534 site-years from 1980 to 2014 were used. Mortality from tetanus was estimated using the Cause of Death Ensemble modeling strategy.

**Results:**

There were 56,743 (95% uncertainty interval (UI): 48,199 to 80,042) deaths due to tetanus in 2015; 19,937 (UI: 17,021 to 23,467) deaths occurred in neonates; and 36,806 (UI: 29,452 to 61,481) deaths occurred in older children and adults. Of the 19,937 neonatal tetanus deaths, 45% of deaths occurred in South Asia, and 44% in Sub-Saharan Africa. Of the 36,806 deaths after the neonatal period, 47% of deaths occurred in South Asia, 36% in sub-Saharan Africa, and 12% in Southeast Asia. Between 1990 and 2015, the global mortality rate due to neonatal tetanus dropped by 90% and that due to non-neonatal tetanus dropped by 81%. However, tetanus mortality rates were still high in a number of countries in 2015. The highest rates of neonatal tetanus mortality (more than 1,000 deaths per 100,000 population) were observed in Somalia, South Sudan, Afghanistan, and Kenya. The highest rates of mortality from tetanus after the neonatal period (more than 5 deaths per 100,000 population) were observed in Somalia, South Sudan, and Kenya.

**Conclusions:**

Though there have been tremendous strides globally in reducing the burden of tetanus, tens of thousands of unnecessary deaths from tetanus could be prevented each year by an already available inexpensive and effective vaccine. Availability of more high quality data could help narrow the uncertainty of tetanus mortality estimates.

**Electronic supplementary material:**

The online version of this article (doi:10.1186/s12889-017-4111-4) contains supplementary material, which is available to authorized users.

## Background

Tetanus, commonly referred to as “lockjaw”, is a serious infection caused by *Clostridium tetani*. The bacterium is commonly found in the environment (usually in soil, dust, and animal waste). Tetanus spores can enter the body through cuts or abrasions. Newborns can become infected through contaminated instruments used to cut the umbilical cord or by improper handling of the umbilical stump [1]. Neonatal tetanus is more likely to occur in low and middle income countries especially in places such as urban slums and rural areas; in those places unhygienic deliveries at home are common, and coverage of antenatal care services and maternal tetanus toxoid immunization are usually inadequate [2–4].

During the past two decades, there has been a dramatic decline in tetanus cases and deaths due to the scale up of immunization programs [5, 6]. Despite the availability of an inexpensive and effective tetanus vaccine, many people in low and middle income countries continue to die from tetanus. In developed countries, tetanus is rare but occasional cases and deaths continue to occur in unvaccinated individuals. The current patterns and distribution of tetanus mortality have not been well documented. In this study, we identify the global, regional and national levels and trends of neonatal and non-neonatal tetanus mortality between 1990 and 2015, based on the findings from the Global Burden of Disease Study 2015.

## Methods

Data from vital registration, verbal autopsy, and mortality surveillance data covering 12,534 site-years from 1980 to 2014 were used for this study [7]. The International Classification of Diseases (ICD) codes for neonatal tetanus include ICD-10 codes (A33-A35.0) and ICD-9 codes (037–037.9, 771.3). Further details about data sources are provided in the Web Appendix. We used the Cause of Death Ensemble model (CODEm) strategy [7–10], which has been widely used for generating global estimates of cause-specific mortality. The CODEm strategy evaluates potential models that apply different functional forms (mixed effects models and space-time Gaussian Process Regression models) to mortality rates or cause fractions with varying combinations of predictive covariates [7], including DTP3 coverage proportion, educational attainment, health system access, in-facility delivery proportion, lagged distributed income, skilled birth attendance proportion, and tetanus toxoid coverage proportion. An ensemble of models that performs best on out-of-sample predictive validity tests was then selected as the best model. A complete time series of the parameters for each covariate for each location was estimated using data from household surveys, censuses, official reports, administrative data, and systematic reviews. The sources and imputation methods used to generate time series for the covariates have been published elsewhere [11].

## Results

**Fig. 1:**
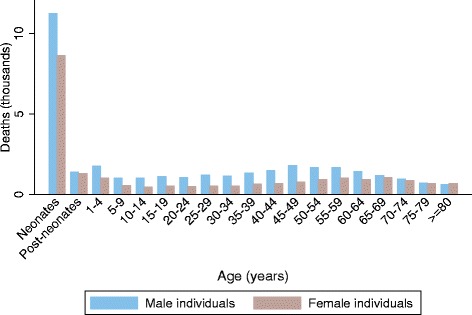
Global age-sex distribution of tetanus deaths in 2015

There were 56,743 (95% uncertainty interval (UI): 48,199 to 80,042) deaths due to tetanus in 2015: 19,937 (UI: 17,021 to 23,467) deaths occurred in neonates and 36,806 (UI: 29,452 to 61,481) deaths occurred after the neonatal period (Table 1). Of all neonatal tetanus deaths, 45% of deaths occurred in South Asia. Sub-Saharan Africa accounted for additional 44% of deaths; 67% of these deaths occurred in eastern sub-Saharan Africa, 27% in western sub-Saharan Africa, and 6% in central sub-Saharan Africa. Of tetanus deaths after the neonatal period, 47% of deaths occurred in South Asia, 36% in sub-Saharan Africa, and 12% in Southeast Asia. Figure 1 shows the global age-sex distribution of tetanus mortality in 2015. Tetanus deaths were concentrated in neonates when they were compared with deaths in each of the other age categories (Fig. 1). More deaths occurred in males than females in most age groups (Fig. 1). Age-standardized tetanus mortality rate (per 100,000 people) among males (0.93, UI: 0.72 to 1.44) was also higher than that among females (0.63, UI: 0.50 to 0.90) (data not shown).

**Table 1:** Neonatal and non-neonatal tetanus deaths, mortality rates, and change in mortality rates between 1990 and 2015 for 21 Global Burden of Disease regions and 195 countries and territories (Regions ranked from the highest to lowest neonatal mortality rate in 2015)

	1990				2015				% change in rate between 1990 and 2015	
	Deaths (UI)		Rate per 100,000 (UI)		Deaths (UI)		Rate per 100,000 (UI)			
	Neonatal tetanus	Non-neonatal tetanus	Neonatal tetanus	Non-neonatal tetanus	Neonatal tetanus	Non-neonatal tetanus	Neonatal tetanus	Non-neonatal tetanus	Neonatal tetanus	Non-neonatal tetanus
*Global*	199,118 (176,424 to 228,139)	137,904 (118,003 to 201,898)	1,919.00 (1,700.28 to 2,198.69)	2.61 (2.23 to 3.81)	19,937 (17,021 to 23,467)	36,806 (29,452 to 61,481)	187.47 (160.05 to 220.66)	0.50 (0.40 to 0.84)	−90.23 (−91.73 to −88.48)	−80.81 (−84.12 to −75.28)
*Eastern Sub-Saharan Africa*	16,642 (13,308 to 22,798)	10,775 (7,511 to 15,087)	2,610.92 (2,087.93 to 3,576.80)	5.81 (4.05 to 8.14)	5,949 (4,562 to 8,831)	8,834 (5,532 to 13,689)	577.12 (442.52 to 856.74)	2.35 (1.47 to 3.64)	−77.90 (−82.94 to −70.89)	−59.56 (−69.85 to −44.70)
Burundi	505 (262 to 858)	333 (170 to 557)	2,441.79 (1,268.06 to 4,149.11)	5.93 (3.04 to 9.93)	279 (134 to 529)	290 (98 to 639)	762.11 (364.89 to 1,444.51)	2.59 (0.87 to 5.70)	−68.79 (−85.82 to −31.04)	−56.31 (−82.33 to 3.79)
Comoros	33 (18 to 54)	16 (8 to 31)	2,494.51 (1,351.41 to 4,112.77)	3.81 (1.82 to 7.50)	6 (3 to 11)	13 (6 to 27)	317.14 (154.22 to 572.90)	1.63 (0.76 to 3.38)	−87.29 (−94.51 to −72.59)	−57.07 (−80.45 to −7.95)
Djibouti	16 (4 to 33)	12 (4 to 27)	950.85 (244.13 to 1,927.05)	2.06 (0.66 to 4.74)	8 (3 to 16)	16 (5 to 43)	496.44 (185.53 to 945.90)	1.84 (0.54 to 4.88)	−47.79 (−76.03 to 31.37)	−10.64 (−65.63 to 130.12)
Eritrea	519 (256 to 919)	325 (193 to 528)	5,265.69 (2,595.33 to 9,315.49)	10.37 (6.16 to 16.87)	65 (31 to 121)	129 (41 to 263)	490.80 (236.72 to 917.28)	2.46 (0.78 to 5.03)	−90.68 (−96.11 to −77.41)	−76.23 (−93.75 to −43.45)
Ethiopia	3,574 (2,202 to 5,470)	2,508 (1,448 to 3,803)	2,123.05 (1,308.21 to 3,249.19)	5.26 (3.03 to 7.97)	736 (389 to 1,247)	1,238 (601 to 2,358)	308.09 (162.93 to 522.16)	1.25 (0.61 to 2.38)	−85.49 (−93.06 to −72.08)	−76.25 (−88.05 to −54.49)
Kenya	2,887 (1,753 to 5,846)	2,089 (851 to 4,743)	3,894.64 (2,365.00 to 7,885.42)	8.94 (3.64 to 20.30)	1,391 (921 to 2,601)	2,592 (927 to 5,662)	1,173.55 (776.38 to 2,193.85)	5.63 (2.01 to 12.29)	−69.87 (−77.35 to −55.04)	−37.06 (−57.26 to −9.41)
Madagascar	492 (349 to 679)	261 (184 to 359)	1,256.61 (891.69 to 1,736.04)	2.27 (1.60 to 3.12)	83 (36 to 184)	232 (74 to 548)	132.90 (58.18 to 293.54)	0.96 (0.31 to 2.27)	−89.42 (−95.51 to −74.41)	−57.63 (−86.46 to −2.15)
Malawi	655 (382 to 1,056)	292 (152 to 509)	1,984.98 (1,158.35 to 3,198.51)	3.11 (1.62 to 5.44)	147 (75 to 257)	244 (101 to 612)	293.77 (149.48 to 514.35)	1.42 (0.59 to 3.57)	−85.20 (−93.32 to −68.05)	−54.44 (−76.14 to −14.58)
Mozambique	1,175 (602 to 2,171)	997 (493 to 1,728)	2,537.78 (1,301.84 to 4,690.31)	7.43 (3.67 to 12.88)	127 (67 to 237)	387 (143 to 710)	155.53 (82.14 to 290.88)	1.39 (0.51 to 2.54)	−93.87 (−97.47 to −85.31)	−81.35 (−91.88 to −56.71)
Rwanda	396 (216 to 681)	259 (109 to 456)	1,658.13 (902.82 to 2,846.36)	3.59 (1.51 to 6.31)	123 (65 to 214)	139 (55 to 285)	452.85 (239.79 to 784.94)	1.20 (0.48 to 2.45)	−72.69 (−86.82 to −40.58)	−66.63 (−83.38 to −32.66)
Somalia	1,441 (863 to 2,388)	1,171 (421 to 2,695)	6,423.06 (3,846.02 to 10,645.96)	18.36 (6.61 to 42.25)	1,188 (609 to 2,269)	1,114 (384 to 2,563)	3,376.39 (1,731.55 to 6,447.88)	10.30 (3.55 to 23.70)	−47.43 (−71.98 to −5.76)	−43.90 (−79.37 to 70.70)
South Sudan	787 (211 to 1,637)	443 (90 to 1,225)	3,985.03 (1,069.25 to 8,290.52)	7.63 (1.56 to 21.10)	668 (163 to 1,540)	934 (133 to 3,693)	2,002.54 (488.52 to 4,612.55)	7.62 (1.09 to 30.14)	−49.75 (−77.23 to 1.73)	−0.12 (−71.92 to 186.62)
Tanzania	1,001 (603 to 1,539)	776 (504 to 1,109)	1,192.20 (717.90 to 1,832.84)	3.06 (1.99 to 4.37)	488 (273 to 856)	683 (334 to 1,314)	313.72 (175.59 to 550.25)	1.28 (0.63 to 2.47)	−73.69 (−87.63 to −42.73)	−58.03 (−80.13 to −15.10)
Uganda	2,231 (1,288 to 3,760)	850 (521 to 1,344)	3,461.47 (1,997.42 to 5,833.30)	4.90 (3.00 to 7.74)	424 (243 to 705)	432 (234 to 758)	338.77 (193.95 to 562.94)	1.11 (0.60 to 1.94)	−90.21 (−95.53 to −79.70)	−77.40 (−89.15 to −54.21)
Zambia	921 (480 to 1,519)	439 (245 to 787)	3,277.63 (1,709.24 to 5,405.20)	5.42 (3.03 to 9.71)	211 (123 to 341)	385 (202 to 676)	434.25 (251.90 to 700.03)	2.38 (1.24 to 4.17)	−86.75 (−93.16 to −71.83)	−56.12 (−81.40 to −2.09)
*South Asia*	130,517 (116,984 to 146,106)	90,694 (75,558 to 147,756)	4,890.61 (4,383.52 to 5,474.75)	8.24 (6.86 to 13.42)	8,922 (6,735 to 11,932)	17,444 (12,964 to 31,854)	340.41 (256.98 to 455.26)	1.03 (0.77 to 1.89)	−93.04 (−94.78 to −90.69)	−87.45 (−90.25 to −83.10)
Bangladesh	29,102 (22,276 to 37,832)	4,464 (2,615 to 6,323)	10,572.00 (8,092.31 to 13,743.51)	4.22 (2.47 to 5.98)	1,047 (576 to 1,700)	701 (186 to 1,049)	442.94 (243.61 to 719.28)	0.44 (0.12 to 0.65)	−95.81 (−97.71 to −92.91)	−89.67 (−96.22 to −83.04)
Bhutan	83 (42 to 139)	48 (22 to 93)	5,771.54 (2,924.79 to 9,666.94)	8.93 (4.13 to 17.42)	4 (1 to 9)	8 (2 to 21)	402.81 (107.83 to 885.78)	0.99 (0.24 to 2.69)	−93.02 (−97.55 to −85.33)	−88.89 (−96.55 to −74.27)
India	78,017 (70,753 to 85,683)	77,848 (64,707 to 134,650)	3,861.91 (3,502.32 to 4,241.36)	8.96 (7.45 to 15.50)	6,079 (4,350 to 8,304)	14,720 (10,159 to 30,040)	314.21 (224.85 to 429.24)	1.12 (0.78 to 2.29)	−91.86 (−94.33 to −88.35)	−87.46 (−90.82 to −82.23)
Nepal	7,519 (4,586 to 11,519)	3,561 (1,835 to 5,905)	14,104.21 (8,601.59 to 21,606.63)	19.07 (9.82 to 31.61)	339 (188 to 568)	402 (215 to 563)	778.52 (432.88 to 1,305.61)	1.41 (0.75 to 1.97)	−94.48 (−97.07 to −89.17)	−92.60 (−96.18 to −84.12)
Pakistan	15,796 (9,396 to 24,277)	4,773 (3,139 to 7,184)	4,958.99 (2,949.79 to 7,621.60)	4.44 (2.92 to 6.68)	1,453 (823 to 2,423)	1,613 (1,039 to 2,332)	358.50 (202.96 to 597.71)	0.86 (0.55 to 1.24)	−92.77 (−96.41 to −85.29)	−80.73 (−88.44 to −69.19)
*Western Sub-Saharan Africa*	13,791 (10,062 to 18,136)	7,379 (4,478 to 10,861)	2,097.30 (1,530.17 to 2,758.02)	3.73 (2.26 to 5.48)	2,337 (1,710 to 3,216)	2,928 (1,865 to 5,790)	206.50 (151.05 to 284.13)	0.75 (0.48 to 1.48)	−90.15 (−93.51 to −83.46)	−79.85 (−87.35 to −63.09)
Benin	282 (147 to 484)	155 (82 to 248)	1,635.25 (849.37 to 2,800.77)	3.11 (1.64 to 4.97)	33 (15 to 65)	64 (29 to 161)	113.76 (52.23 to 224.95)	0.59 (0.27 to 1.48)	−93.04 (−97.26 to −81.06)	−81.08 (−91.89 to −45.57)
Burkina Faso	1,012 (547 to 1,712)	503 (296 to 861)	3,259.31 (1,763.06 to 5,512.58)	5.72 (3.37 to 9.81)	178 (86 to 325)	182 (95 to 310)	331.78 (160.36 to 605.15)	1.01 (0.52 to 1.72)	−89.82 (−96.07 to −74.45)	−82.37 (−93.05 to −64.18)
Cameroon	522 (183 to 1,086)	309 (137 to 665)	1,298.98 (455.17 to 2,700.69)	2.56 (1.14 to 5.52)	53 (22 to 108)	82 (26 to 197)	83.76 (34.42 to 171.03)	0.35 (0.11 to 0.84)	−93.55 (−97.52 to −80.26)	−86.31 (−95.20 to −52.00)
Cape Verde	0 (0 to 1)	1 (0 to 2)	40.92 (17.36 to 86.20)	0.26 (0.05 to 0.53)	0 (0 to 0)	0 (0 to 0)	1.33 (0.69 to 2.39)	0.03 (0.02 to 0.05)	−96.75 (−98.71 to −91.34)	−87.80 (−94.41 to −43.82)
Chad	1,060 (519 to 1,833)	583 (280 to 1,218)	4,714.89 (2,307.87 to 8,148.41)	9.82 (4.72 to 20.52)	389 (196 to 714)	258 (105 to 667)	828.34 (417.34 to 1,520.79)	1.84 (0.75 to 4.76)	−82.43 (−92.28 to −60.66)	−81.28 (−93.16 to −53.33)
Cote d’Ivoire	594 (294 to 1,037)	321 (132 to 588)	1,537.12 (759.69 to 2,683.53)	2.65 (1.09 to 4.85)	121 (60 to 223)	107 (55 to 222)	193.52 (96.85 to 357.66)	0.47 (0.25 to 0.98)	−87.41 (−94.39 to −69.62)	−82.11 (−92.32 to −40.88)
Ghana	676 (362 to 1,142)	569 (241 to 931)	1,590.87 (851.82 to 2,690.05)	3.90 (1.65 to 6.38)	145 (83 to 242)	175 (86 to 332)	218.17 (124.19 to 364.90)	0.64 (0.32 to 1.21)	−86.29 (−93.54 to −67.58)	−83.62 (−92.12 to −62.17)
Guinea	1,389 (567 to 2,383)	336 (149 to 656)	6,577.60 (2,686.19 to 11,288.25)	5.59 (2.47 to 10.91)	178 (99 to 301)	165 (73 to 364)	519.16 (289.77 to 877.50)	1.32 (0.58 to 2.90)	−92.11 (−96.48 to −79.48)	−76.38 (−90.58 to −38.87)
Guinea-Bissau	82 (47 to 133)	43 (18 to 89)	2,325.61 (1,341.01 to 3,759.28)	4.08 (1.75 to 8.48)	17 (9 to 31)	18 (6 to 46)	345.73 (171.06 to 609.87)	0.96 (0.32 to 2.51)	−85.13 (−93.40 to −67.02)	−76.43 (−92.52 to −15.97)
Liberia	125 (74 to 205)	60 (30 to 110)	1,780.95 (1,048.11 to 2,915.55)	2.84 (1.42 to 5.16)	12 (4 to 32)	49 (13 to 208)	99.04 (36.66 to 271.86)	1.08 (0.29 to 4.63)	−94.44 (−97.99 to −83.74)	−61.81 (−89.48 to 28.68)
Mali	90 (39 to 177)	522 (224 to 856)	295.43 (127.84 to 583.13)	6.16 (2.64 to 10.10)	18 (2 to 67)	125 (55 to 318)	31.64 (3.90 to 120.05)	0.71 (0.32 to 1.81)	−89.29 (−97.77 to −67.92)	−88.41 (−95.16 to −64.89)
Mauritania	86 (45 to 149)	80 (20 to 235)	1,407.14 (741.30 to 2,430.10)	3.94 (1.00 to 11.60)	11 (6 to 21)	14 (5 to 33)	113.83 (57.85 to 209.23)	0.33 (0.13 to 0.80)	−91.91 (−96.55 to −81.26)	−91.52 (−97.08 to −62.30)
Niger	1,645 (881 to 2,844)	821 (426 to 1,354)	5,075.00 (2,716.96 to 8,771.49)	10.42 (5.40 to 17.17)	259 (114 to 584)	257 (89 to 713)	349.83 (154.57 to 789.57)	1.30 (0.45 to 3.61)	−93.11 (−97.45 to −82.80)	−87.52 (−96.61 to −48.72)
Nigeria	5,550 (3,304 to 8,734)	2,639 (1,500 to 4,451)	1,784.94 (1,062.80 to 2,809.18)	2.76 (1.57 to 4.66)	799 (398 to 1,575)	1,273 (445 to 3,536)	150.13 (74.72 to 295.88)	0.70 (0.24 to 1.94)	−91.59 (−96.29 to −80.75)	−74.70 (−91.15 to −41.90)
Sao Tome and Principe	2 (1 to 3)	10 (6 to 16)	574.98 (315.21 to 981.36)	8.81 (4.95 to 14.07)	0 (0 to 1)	6 (2 to 13)	70.83 (33.48 to 140.46)	2.97 (0.91 to 6.60)	−87.68 (−94.99 to −70.43)	−66.32 (−87.36 to −32.88)
Senegal	375 (219 to 628)	192 (94 to 324)	1,535.95 (898.48 to 2,571.63)	2.57 (1.25 to 4.33)	51 (25 to 94)	64 (27 to 164)	118.94 (57.82 to 220.05)	0.42 (0.18 to 1.09)	−92.26 (−96.71 to −82.52)	−83.56 (−93.24 to −45.87)
Sierra Leone	147 (62 to 291)	124 (56 to 216)	1,133.98 (476.89 to 2,244.16)	3.16 (1.42 to 5.52)	36 (17 to 65)	39 (16 to 106)	208.36 (99.18 to 382.15)	0.61 (0.25 to 1.65)	−81.63 (−92.00 to −58.50)	−80.65 (−91.86 to −45.89)
The Gambia	24 (16 to 36)	13 (7 to 21)	747.87 (499.97 to 1,110.26)	1.37 (0.75 to 2.30)	12 (6 to 24)	11 (5 to 23)	186.64 (88.62 to 386.26)	0.54 (0.24 to 1.14)	−75.04 (−89.31 to −43.69)	−60.59 (−82.40 to −21.28)
Togo	129 (72 to 221)	100 (57 to 145)	1,078.12 (601.66 to 1,851.57)	2.64 (1.51 to 3.85)	26 (14 to 52)	40 (16 to 125)	137.58 (70.52 to 270.39)	0.55 (0.22 to 1.72)	−87.24 (−94.49 to −70.43)	−79.12 (−92.73 to −19.23)
*Central Sub-Saharan Africa*	1,979 (1,272 to 2,959)	1,434 (498 to 3,178)	1,046.37 (672.61 to 1,564.55)	2.71 (0.94 to 6.01)	530 (294 to 934)	1,279 (297 to 5,328)	148.54 (82.32 to 261.74)	1.12 (0.26 to 4.66)	−85.80 (−93.52 to −69.43)	−58.78 (−87.86 to 27.27)
Angola	704 (305 to 1,475)	380 (92 to 1,240)	1,602.73 (694.73 to 3,358.72)	3.39 (0.82 to 11.06)	69 (16 to 153)	91 (15 to 342)	81.54 (19.08 to 180.26)	0.36 (0.06 to 1.36)	−94.91 (−98.17 to −87.48)	−89.29 (−97.56 to −62.20)
Central African Republic	132 (69 to 224)	101 (37 to 205)	1,471.61 (770.47 to 2,506.44)	3.44 (1.26 to 7.00)	62 (29 to 117)	136 (32 to 391)	509.74 (234.85 to 961.54)	2.79 (0.66 to 7.99)	−65.36 (−85.66 to −17.47)	−18.93 (−69.90 to 83.44)
Congo	9 (3 to 19)	20 (4 to 45)	138.02 (44.52 to 282.70)	0.82 (0.19 to 1.87)	4 (2 to 10)	14 (3 to 42)	32.73 (15.16 to 77.50)	0.30 (0.06 to 0.90)	−76.28 (−90.97 to −26.78)	−63.03 (−82.97 to −23.16)
Democratic Republic of the Congo	1,122 (651 to 1,792)	920 (295 to 2,179)	894.53 (518.84 to 1,428.11)	2.63 (0.84 to 6.22)	392 (165 to 769)	1,031 (185 to 4,899)	162.40 (68.41 to 318.96)	1.34 (0.24 to 6.35)	−81.85 (−93.49 to −56.66)	−49.13 (−87.89 to 67.57)
Equatorial Guinea	9 (5 to 17)	9 (3 to 23)	705.17 (357.03 to 1,294.33)	2.51 (0.81 to 6.05)	2 (0 to 7)	3 (0 to 19)	94.98 (16.65 to 323.58)	0.34 (0.02 to 2.26)	−86.53 (−97.96 to −48.97)	−86.27 (−99.07 to −12.86)
Gabon	3 (0 to 6)	5 (0 to 15)	95.94 (18.00 to 243.98)	0.55 (0.05 to 1.61)	1 (0 to 3)	4 (0 to 17)	23.19 (6.24 to 85.47)	0.21 (0.02 to 0.97)	−75.83 (−91.07 to −35.20)	−61.35 (−83.48 to −19.87)
*North Africa and Middle East*	10,817 (6,911 to 16,193)	2,379 (1,513 to 3,773)	1,275.80 (815.17 to 1,909.97)	0.71 (0.45 to 1.13)	1,364 (802 to 2,226)	541 (377 to 819)	134.08 (78.87 to 218.82)	0.10 (0.07 to 0.14)	−89.49 (−94.06 to −82.62)	−86.51 (−92.36 to −76.45)
Afghanistan	6,006 (2,430 to 10,493)	1,075 (309 to 2,380)	12,935.74 (5,233.49 to 22,598.70)	8.77 (2.52 to 19.42)	1,260 (702 to 2,117)	289 (126 to 565)	1,557.12 (867.55 to 2,616.03)	0.89 (0.39 to 1.74)	−87.96 (−94.21 to −73.64)	−89.85 (−96.61 to −59.39)
Algeria	93 (37 to 182)	44 (15 to 97)	148.02 (59.58 to 290.16)	0.17 (0.06 to 0.37)	6 (3 to 12)	7 (5 to 10)	9.02 (4.18 to 17.13)	0.02 (0.01 to 0.03)	−93.91 (−97.67 to −81.64)	−88.98 (−95.42 to −68.73)
Bahrain	0 (0 to 0)	0 (0 to 0)	6.95 (3.39 to 10.30)	0.03 (0.02 to 0.04)	0 (0 to 0)	0 (0 to 0)	0.18 (0.12 to 0.27)	0.01 (0.01 to 0.01)	−97.35 (−98.44 to −94.08)	−62.12 (−73.46 to −30.06)
Egypt	882 (568 to 1,351)	458 (251 to 566)	627.55 (403.83 to 961.23)	0.82 (0.45 to 1.01)	19 (10 to 31)	85 (62 to 189)	9.87 (5.47 to 16.40)	0.09 (0.07 to 0.21)	−98.43 (−99.22 to −96.92)	−88.51 (−92.49 to −68.67)
Iran	100 (45 to 188)	55 (32 to 99)	72.33 (32.81 to 136.50)	0.10 (0.06 to 0.18)	2 (1 to 3)	19 (12 to 28)	1.52 (0.74 to 2.89)	0.02 (0.02 to 0.04)	−97.89 (−99.12 to −94.37)	−75.32 (−88.34 to −51.98)
Iraq	26 (11 to 50)	12 (6 to 23)	51.47 (22.42 to 100.64)	0.07 (0.04 to 0.13)	4 (2 to 9)	12 (7 to 20)	4.74 (2.35 to 9.05)	0.03 (0.02 to 0.06)	−90.79 (−96.47 to −72.95)	−51.21 (−79.13 to −9.39)
Jordan	0 (0 to 1)	0 (0 to 1)	3.38 (1.64 to 6.06)	0.01 (0.01 to 0.02)	0 (0 to 0)	1 (1 to 1)	0.16 (0.09 to 0.27)	0.01 (0.01 to 0.01)	−95.25 (−97.86 to −87.86)	−26.10 (−41.27 to −8.49)
Kuwait	0 (0 to 0)	0 (0 to 0)	1.09 (0.72 to 1.57)	0.01 (0.00 to 0.01)	0 (0 to 0)	0 (0 to 0)	0.12 (0.08 to 0.16)	0.00 (0.00 to 0.00)	−89.18 (−93.30 to −81.34)	−87.73 (−92.00 to −81.59)
Lebanon	0 (0 to 1)	1 (1 to 1)	9.10 (4.23 to 16.39)	0.03 (0.02 to 0.04)	0 (0 to 0)	1 (1 to 1)	0.36 (0.13 to 0.99)	0.01 (0.01 to 0.02)	−95.99 (−98.83 to −87.68)	−54.21 (−70.50 to −26.10)
Libya	3 (1 to 6)	2 (1 to 3)	27.28 (7.22 to 60.38)	0.04 (0.02 to 0.07)	0 (0 to 0)	1 (1 to 1)	2.59 (1.22 to 4.95)	0.02 (0.01 to 0.02)	−90.51 (−96.39 to −69.15)	−57.49 (−77.89 to −14.04)
Morocco	898 (538 to 1,398)	99 (57 to 159)	1,627.61 (974.97 to 2,533.64)	0.40 (0.23 to 0.63)	3 (2 to 6)	24 (14 to 45)	6.17 (3.44 to 10.39)	0.07 (0.04 to 0.13)	−99.62 (−99.82 to −99.21)	−82.50 (−91.01 to −65.29)
Oman	1 (0 to 3)	1 (0 to 1)	21.52 (6.85 to 60.75)	0.04 (0.02 to 0.07)	0 (0 to 0)	1 (1 to 1)	1.05 (0.50 to 1.89)	0.02 (0.01 to 0.02)	−95.13 (−98.68 to −79.74)	−54.20 (−76.14 to −23.72)
Palestine	0 (0 to 0)	0 (0 to 0)	1.93 (0.84 to 3.46)	0.02 (0.01 to 0.02)	0 (0 to 0)	1 (0 to 1)	0.18 (0.09 to 0.32)	0.01 (0.01 to 0.01)	−90.56 (−96.29 to −70.14)	−24.43 (−43.24 to −0.51)
Qatar	0 (0 to 0)	0 (0 to 0)	1.86 (0.86 to 3.50)	0.01 (0.01 to 0.02)	0 (0 to 0)	0 (0 to 0)	0.12 (0.06 to 0.22)	0.01 (0.01 to 0.01)	−93.58 (−97.51 to −82.80)	−16.00 (−39.30 to 12.28)
Saudi Arabia	18 (7 to 37)	14 (6 to 30)	41.89 (15.79 to 83.76)	0.09 (0.04 to 0.19)	1 (1 to 2)	7 (4 to 9)	2.19 (1.22 to 3.69)	0.02 (0.01 to 0.03)	−94.78 (−97.95 to −82.45)	−75.55 (−90.15 to −42.66)
Sudan	335 (133 to 644)	124 (38 to 254)	529.66 (211.04 to 1,019.34)	0.62 (0.19 to 1.27)	30 (13 to 57)	25 (10 to 50)	30.11 (12.82 to 57.79)	0.06 (0.03 to 0.13)	−94.32 (−97.57 to −86.12)	−90.18 (−95.07 to −77.97)
Syria	234 (135 to 373)	37 (17 to 71)	694.90 (400.52 to 1,108.49)	0.30 (0.14 to 0.57)	2 (1 to 3)	5 (3 to 8)	4.94 (2.68 to 8.43)	0.03 (0.02 to 0.04)	−99.29 (−99.67 to −98.54)	−91.40 (−95.78 to −79.29)
Tunisia	2 (1 to 3)	3 (1 to 5)	10.74 (4.11 to 20.10)	0.04 (0.02 to 0.06)	0 (0 to 0)	2 (1 to 3)	0.13 (0.08 to 0.23)	0.02 (0.01 to 0.03)	−98.75 (−99.49 to −96.39)	−40.39 (−66.86 to 8.12)
Turkey	1,477 (811 to 2,417)	241 (114 to 475)	1,398.68 (768.29 to 2,289.23)	0.44 (0.21 to 0.87)	3 (1 to 5)	14 (11 to 29)	2.81 (1.50 to 4.64)	0.02 (0.01 to 0.04)	−99.80 (−99.92 to −99.57)	−95.88 (−98.04 to −90.77)
United Arab Emirates	4 (1 to 11)	8 (4 to 14)	117.59 (26.52 to 303.85)	0.46 (0.23 to 0.75)	0 (0 to 1)	25 (15 to 45)	3.63 (1.22 to 8.93)	0.28 (0.17 to 0.49)	−96.91 (−99.30 to −75.06)	−39.46 (−70.49 to 47.29)
Yemen	731 (317 to 1,710)	205 (75 to 476)	1,549.33 (672.76 to 3,624.06)	1.72 (0.63 to 3.99)	32 (16 to 60)	21 (10 to 41)	49.56 (24.57 to 93.33)	0.08 (0.04 to 0.15)	−96.80 (−98.83 to −91.46)	−95.35 (−98.45 to −83.92)
*Caribbean*	416 (236 to 679)	310 (145 to 526)	614.45 (348.58 to 1,003.59)	0.87 (0.41 to 1.47)	48 (23 to 101)	107 (38 to 360)	80.30 (39.02 to 170.14)	0.24 (0.08 to 0.80)	−86.93 (−94.99 to −65.08)	−72.70 (−86.46 to −15.16)
Antigua and Barbuda	0 (0 to 0)	0 (0 to 0)	31.99 (22.34 to 43.90)	0.31 (0.27 to 0.35)	0 (0 to 0)	0 (0 to 0)	2.32 (1.44 to 3.75)	0.02 (0.01 to 0.02)	−92.73 (−95.87 to −86.97)	−94.68 (−95.85 to −92.43)
Barbados	0 (0 to 0)	1 (1 to 1)	60.34 (38.92 to 90.25)	0.46 (0.41 to 0.53)	0 (0 to 0)	0 (0 to 0)	3.47 (1.83 to 5.84)	0.04 (0.03 to 0.06)	−94.25 (−97.25 to −88.64)	−90.32 (−92.67 to −86.03)
Belize	0 (0 to 1)	1 (0 to 1)	92.67 (63.37 to 133.29)	0.41 (0.17 to 0.52)	0 (0 to 0)	0 (0 to 1)	3.29 (1.74 to 5.79)	0.05 (0.03 to 0.17)	−96.45 (−98.34 to −93.04)	−88.21 (−94.27 to −38.40)
Bermuda	0 (0 to 0)	0 (0 to 0)	4.26 (2.73 to 6.42)	0.07 (0.06 to 0.07)	0 (0 to 0)	0 (0 to 0)	0.23 (0.12 to 0.40)	0.00 (0.00 to 0.01)	−94.58 (−97.40 to −89.70)	−93.78 (−95.13 to −92.03)
Cuba	0 (0 to 0)	2 (2 to 3)	1.06 (0.87 to 1.29)	0.02 (0.02 to 0.03)	0 (0 to 0)	0 (0 to 0)	0.15 (0.11 to 0.20)	0.00 (0.00 to 0.00)	−86.29 (−90.24 to −80.40)	−89.23 (−91.18 to −84.22)
Dominica	0 (0 to 0)	1 (0 to 1)	98.14 (67.41 to 140.95)	0.77 (0.36 to 0.94)	0 (0 to 0)	0 (0 to 0)	9.84 (5.64 to 16.46)	0.12 (0.06 to 0.46)	−89.97 (−94.75 to −80.51)	−84.94 (−93.20 to −13.39)
Dominican Republic	14 (10 to 18)	30 (23 to 50)	85.97 (64.23 to 112.33)	0.42 (0.32 to 0.70)	4 (3 to 6)	22 (12 to 33)	26.42 (17.39 to 38.60)	0.21 (0.12 to 0.31)	−69.27 (−81.85 to −51.14)	−50.76 (−75.30 to −25.87)
Grenada	0 (0 to 0)	1 (0 to 1)	76.71 (42.97 to 121.19)	0.65 (0.30 to 0.79)	0 (0 to 0)	0 (0 to 0)	3.37 (1.59 to 6.29)	0.07 (0.04 to 0.25)	−95.61 (−98.17 to −89.67)	−88.74 (−94.30 to −38.39)
Guyana	1 (1 to 2)	2 (1 to 3)	67.75 (48.80 to 93.88)	0.33 (0.15 to 0.40)	0 (0 to 0)	0 (0 to 1)	4.73 (3.11 to 7.24)	0.06 (0.03 to 0.13)	−93.02 (−95.98 to −88.02)	−82.67 (−90.44 to −40.57)
Haiti	384 (210 to 637)	248 (93 to 457)	1,947.17 (1,065.07 to 3,231.50)	3.50 (1.32 to 6.45)	42 (18 to 93)	77 (11 to 315)	209.26 (90.08 to 469.31)	0.72 (0.10 to 2.95)	−89.25 (−96.28 to −69.12)	−79.34 (−95.21 to −21.30)
Jamaica	3 (2 to 4)	5 (2 to 6)	64.21 (41.80 to 94.09)	0.21 (0.10 to 0.26)	0 (0 to 0)	1 (1 to 4)	3.08 (1.71 to 5.13)	0.04 (0.02 to 0.14)	−95.21 (−97.55 to −90.87)	−82.25 (−91.80 to −5.90)
Puerto Rico	0 (0 to 0)	2 (2 to 2)	0.88 (0.75 to 1.03)	0.05 (0.05 to 0.06)	0 (0 to 0)	1 (0 to 1)	0.15 (0.11 to 0.21)	0.01 (0.01 to 0.02)	−82.97 (−87.58 to −75.89)	−73.30 (−80.15 to −62.23)
Saint Lucia	0 (0 to 0)	1 (1 to 1)	80.36 (50.47 to 123.45)	0.62 (0.55 to 0.70)	0 (0 to 0)	0 (0 to 0)	3.68 (1.73 to 6.57)	0.03 (0.02 to 0.05)	−95.41 (−97.97 to −90.00)	−94.76 (−96.22 to −91.88)
Saint Vincent and the Grenadines	0 (0 to 0)	0 (0 to 0)	35.86 (24.47 to 51.04)	0.18 (0.16 to 0.21)	0 (0 to 0)	0 (0 to 0)	1.75 (1.03 to 2.82)	0.02 (0.01 to 0.02)	−95.11 (−97.34 to −90.86)	−91.51 (−93.46 to −87.69)
Suriname	1 (1 to 1)	2 (1 to 2)	113.60 (82.84 to 155.10)	0.47 (0.22 to 0.57)	0 (0 to 0)	0 (0 to 1)	7.17 (4.79 to 10.53)	0.08 (0.04 to 0.27)	−93.69 (−96.18 to −89.11)	−83.70 (−92.41 to −21.04)
The Bahamas	0 (0 to 0)	0 (0 to 0)	19.31 (11.04 to 30.26)	0.10 (0.05 to 0.13)	0 (0 to 0)	0 (0 to 0)	1.65 (0.85 to 3.13)	0.03 (0.02 to 0.07)	−91.45 (−96.17 to −80.42)	−72.99 (−84.92 to −7.31)
Trinidad and Tobago	0 (0 to 0)	3 (2 to 3)	11.24 (8.92 to 14.23)	0.21 (0.19 to 0.23)	0 (0 to 0)	0 (0 to 0)	0.29 (0.16 to 0.49)	0.00 (0.00 to 0.00)	−97.38 (−98.67 to −95.26)	−98.46 (−98.87 to −97.63)
Virgin Islands, U.S.	0 (0 to 0)	0 (0 to 0)	1.95 (1.33 to 2.85)	0.02 (0.01 to 0.02)	0 (0 to 0)	0 (0 to 0)	0.27 (0.18 to 0.41)	0.01 (0.01 to 0.02)	−85.99 (−92.22 to −74.21)	−29.89 (−48.27 to 20.32)
*Southeast Asia*	10,464 (7,157 to 14,800)	14,415 (8,364 to 23,523)	1,118.77 (765.18 to 1,582.32)	3.13 (1.82 to 5.11)	530 (364 to 769)	4,597 (3,124 to 6,522)	57.56 (39.57 to 83.56)	0.71 (0.48 to 1.00)	−94.86 (−96.91 to −91.12)	−77.41 (−86.22 to −56.41)
Cambodia	890 (407 to 1,620)	658 (301 to 1,340)	3,104.97 (1,419.33 to 5,650.98)	7.33 (3.36 to 14.92)	14 (7 to 25)	42 (13 to 113)	49.69 (23.91 to 89.44)	0.27 (0.09 to 0.72)	−98.40 (−99.43 to −94.97)	−96.30 (−98.67 to −91.67)
Indonesia	7,288 (4,168 to 11,357)	8,739 (4,757 to 15,096)	2,069.37 (1,183.43 to 3,224.54)	4.83 (2.63 to 8.34)	406 (240 to 635)	3,559 (2,126 to 5,002)	105.87 (62.72 to 165.62)	1.38 (0.83 to 1.94)	−94.88 (−97.40 to −89.06)	−71.36 (−83.94 to −41.51)
Laos	572 (296 to 980)	405 (202 to 782)	4,276.17 (2,216.13 to 7,325.09)	9.62 (4.79 to 18.56)	15 (7 to 31)	21 (10 to 44)	113.35 (49.65 to 229.19)	0.31 (0.15 to 0.64)	−97.35 (−99.02 to −92.61)	−96.75 (−98.61 to −91.87)
Malaysia	4 (2 to 6)	22 (12 to 30)	9.82 (5.67 to 16.28)	0.12 (0.06 to 0.17)	0 (0 to 0)	12 (8 to 17)	0.62 (0.31 to 1.09)	0.04 (0.03 to 0.06)	−93.72 (−97.32 to −85.12)	−67.21 (−81.64 to −36.60)
Maldives	1 (1 to 2)	1 (0 to 1)	195.02 (110.32 to 332.12)	0.37 (0.18 to 0.62)	0 (0 to 0)	0 (0 to 0)	5.77 (3.33 to 9.87)	0.04 (0.03 to 0.07)	−97.04 (−98.71 to −93.82)	−88.72 (−94.14 to −74.02)
Mauritius	0 (0 to 0)	1 (1 to 1)	9.56 (7.45 to 12.41)	0.06 (0.05 to 0.07)	0 (0 to 0)	0 (0 to 0)	0.32 (0.23 to 0.44)	0.00 (0.00 to 0.00)	−96.64 (−97.86 to −94.94)	−95.84 (−96.57 to −93.51)
Myanmar	517 (197 to 954)	1,821 (312 to 5,948)	612.06 (233.38 to 1,128.90)	4.34 (0.74 to 14.18)	21 (10 to 37)	177 (49 to 546)	29.71 (13.91 to 52.46)	0.33 (0.09 to 1.01)	−95.15 (−97.94 to −88.67)	−92.45 (−96.32 to −79.17)
Philippines	200 (163 to 244)	1,019 (846 to 1,188)	129.45 (105.46 to 158.18)	1.65 (1.37 to 1.92)	47 (31 to 64)	376 (308 to 457)	26.09 (17.63 to 35.85)	0.37 (0.31 to 0.45)	−79.85 (−87.05 to −70.79)	−77.36 (−82.31 to −69.47)
Seychelles	0 (0 to 0)	0 (0 to 0)	32.38 (21.75 to 47.36)	0.44 (0.27 to 0.58)	0 (0 to 0)	0 (0 to 0)	7.38 (4.57 to 11.12)	0.14 (0.09 to 0.27)	−77.22 (−87.62 to −60.99)	−68.70 (−79.65 to −32.35)
Sri Lanka	1 (1 to 2)	6 (5 to 6)	4.97 (4.03 to 5.99)	0.03 (0.03 to 0.04)	1 (0 to 1)	5 (4 to 9)	2.19 (1.38 to 3.43)	0.02 (0.02 to 0.04)	−55.99 (−73.34 to −27.10)	−25.92 (−47.33 to 42.72)
Thailand	127 (87 to 180)	465 (254 to 566)	155.07 (106.80 to 220.44)	0.82 (0.45 to 1.00)	2 (1 to 3)	112 (58 to 342)	3.43 (1.83 to 5.89)	0.16 (0.09 to 0.50)	−97.79 (−98.94 to −95.57)	−79.96 (−90.37 to −16.62)
Timor-Leste	50 (24 to 95)	48 (20 to 103)	2,082.18 (984.44 to 3,898.45)	6.49 (2.64 to 13.73)	1 (0 to 2)	3 (1 to 7)	35.25 (14.96 to 70.58)	0.25 (0.10 to 0.60)	−98.31 (−99.48 to −95.27)	−96.12 (−98.76 to −87.75)
Vietnam	800 (380 to 1,522)	1,210 (609 to 1,971)	540.45 (256.93 to 1,028.69)	1.78 (0.89 to 2.89)	22 (11 to 41)	282 (158 to 467)	18.52 (9.26 to 33.99)	0.30 (0.17 to 0.50)	−96.57 (−98.55 to −91.11)	−82.98 (−91.75 to −54.03)
*East Asia*	13,628 (11,272 to 16,125)	8,651 (4,719 to 10,316)	669.52 (553.78 to 792.19)	0.73 (0.40 to 0.86)	231 (184 to 283)	696 (503 to 1,217)	17.53 (13.95 to 21.50)	0.05 (0.04 to 0.09)	−97.38 (−98.02 to −96.53)	−93.29 (−95.11 to −82.67)
China	13,572 (11,215 to 16,085)	8,621 (4,703 to 10,287)	686.09 (566.96 to 813.15)	0.75 (0.41 to 0.89)	222 (177 to 275)	667 (466 to 1,206)	17.45 (13.90 to 21.54)	0.05 (0.03 to 0.09)	−97.46 (−98.06 to −96.58)	−93.54 (−95.47 to −82.77)
North Korea	56 (20 to 134)	26 (11 to 44)	176.26 (62.36 to 422.93)	0.13 (0.05 to 0.22)	9 (2 to 29)	26 (6 to 66)	31.79 (7.01 to 105.80)	0.10 (0.02 to 0.26)	−81.96 (−96.82 to −7.27)	−20.42 (−80.78 to 208.15)
Taiwan	1 (0 to 1)	4 (3 to 6)	2.32 (1.16 to 4.30)	0.02 (0.01 to 0.03)	0 (0 to 0)	3 (3 to 4)	0.22 (0.11 to 0.40)	0.01 (0.01 to 0.01)	−90.66 (−96.16 to −77.45)	−37.98 (−58.51 to −5.99)
*Oceania*	6 (3 to 12)	5 (2 to 12)	35.91 (14.92 to 73.03)	0.07 (0.03 to 0.18)	2 (1 to 5)	3 (1 to 8)	9.89 (4.12 to 21.44)	0.03 (0.01 to 0.07)	−72.47 (−89.56 to −28.09)	−58.41 (−80.19 to −20.50)
American Samoa	0 (0 to 0)	0 (0 to 0)	1.13 (0.75 to 1.70)	0.01 (0.01 to 0.01)	0 (0 to 0)	0 (0 to 0)	0.18 (0.10 to 0.33)	0.01 (0.01 to 0.01)	−83.95 (−92.21 to −66.08)	−13.13 (−33.34 to 11.26)
Federated States of Micronesia	0 (0 to 0)	0 (0 to 0)	3.31 (1.53 to 6.21)	0.02 (0.01 to 0.04)	0 (0 to 0)	0 (0 to 0)	0.33 (0.09 to 0.91)	0.01 (0.01 to 0.02)	−89.89 (−97.10 to −68.18)	−31.33 (−56.45 to 10.97)
Fiji	0 (0 to 0)	0 (0 to 0)	0.79 (0.45 to 1.27)	0.01 (0.01 to 0.01)	0 (0 to 0)	0 (0 to 0)	0.29 (0.17 to 0.51)	0.01 (0.01 to 0.01)	−62.75 (−83.19 to −16.47)	−1.36 (−25.60 to 28.17)
Guam	0 (0 to 0)	0 (0 to 0)	1.01 (0.70 to 1.49)	0.01 (0.01 to 0.01)	0 (0 to 0)	0 (0 to 0)	0.23 (0.13 to 0.36)	0.01 (0.01 to 0.01)	−77.39 (−87.84 to −58.67)	−4.22 (−21.91 to 16.95)
Kiribati	0 (0 to 0)	0 (0 to 0)	1.99 (1.18 to 3.18)	0.02 (0.02 to 0.03)	0 (0 to 0)	0 (0 to 0)	0.38 (0.14 to 0.91)	0.02 (0.01 to 0.02)	−81.10 (−93.61 to −47.23)	−37.74 (−55.57 to 3.47)
Marshall Islands	0 (0 to 0)	0 (0 to 0)	7.06 (3.33 to 13.97)	0.03 (0.02 to 0.04)	0 (0 to 0)	0 (0 to 0)	1.34 (0.43 to 3.29)	0.01 (0.01 to 0.02)	−80.99 (−94.66 to −40.56)	−46.62 (−67.57 to −19.15)
Northern Mariana Islands	0 (0 to 0)	0 (0 to 0)	1.66 (0.54 to 3.55)	0.01 (0.01 to 0.02)	0 (0 to 0)	0 (0 to 0)	0.13 (0.05 to 0.31)	0.01 (0.01 to 0.01)	−91.88 (−98.08 to −64.84)	−21.43 (−56.36 to 17.88)
Papua New Guinea	5 (2 to 11)	4 (1 to 10)	48.27 (19.21 to 100.03)	0.09 (0.03 to 0.25)	2 (1 to 4)	2 (1 to 6)	12.53 (5.14 to 27.40)	0.03 (0.01 to 0.08)	−74.04 (−90.49 to −29.30)	−62.98 (−83.72 to −20.30)
Samoa	0 (0 to 0)	0 (0 to 0)	3.09 (0.85 to 9.10)	0.02 (0.01 to 0.04)	0 (0 to 0)	0 (0 to 0)	0.63 (0.09 to 2.37)	0.01 (0.01 to 0.02)	−79.75 (−95.44 to −33.53)	−38.66 (−63.81 to −3.37)
Solomon Islands	0 (0 to 0)	0 (0 to 0)	17.03 (6.65 to 45.37)	0.06 (0.02 to 0.15)	0 (0 to 0)	0 (0 to 0)	1.96 (0.73 to 4.72)	0.02 (0.01 to 0.06)	−88.49 (−95.89 to −62.75)	−66.25 (−84.56 to −22.47)
Tonga	0 (0 to 0)	0 (0 to 0)	1.75 (0.83 to 3.38)	0.01 (0.01 to 0.02)	0 (0 to 0)	0 (0 to 0)	0.44 (0.16 to 1.05)	0.01 (0.01 to 0.02)	−74.77 (−91.39 to −31.61)	−16.19 (−42.13 to 19.67)
Vanuatu	0 (0 to 0)	0 (0 to 0)	23.65 (7.59 to 56.07)	0.05 (0.02 to 0.10)	0 (0 to 0)	0 (0 to 0)	2.25 (1.00 to 4.57)	0.02 (0.01 to 0.04)	−90.48 (−96.58 to −70.67)	−62.97 (−83.43 to −16.33)
*Andean Latin America*	28 (21 to 35)	66 (38 to 76)	30.39 (23.54 to 38.45)	0.17 (0.10 to 0.20)	3 (2 to 5)	17 (12 to 37)	3.77 (2.64 to 5.23)	0.03 (0.02 to 0.06)	−87.60 (−91.74 to −81.40)	−83.13 (−88.62 to −59.12)
Bolivia	1 (0 to 2)	3 (1 to 5)	5.18 (2.69 to 9.12)	0.04 (0.01 to 0.07)	0 (0 to 0)	1 (1 to 2)	0.14 (0.07 to 0.23)	0.01 (0.01 to 0.02)	−97.36 (−98.84 to −93.95)	−66.54 (−83.43 to −10.48)
Ecuador	8 (6 to 10)	31 (16 to 36)	34.42 (26.10 to 44.74)	0.30 (0.16 to 0.36)	1 (1 to 2)	7 (4 to 21)	5.43 (3.32 to 8.93)	0.04 (0.03 to 0.13)	−84.22 (−91.25 to −71.92)	−85.77 (−92.00 to −46.99)
Peru	19 (13 to 26)	33 (20 to 40)	37.71 (26.88 to 51.29)	0.15 (0.09 to 0.18)	2 (1 to 3)	8 (6 to 15)	4.36 (2.76 to 6.90)	0.03 (0.02 to 0.05)	−88.43 (−93.13 to −80.27)	−82.17 (−87.53 to −68.83)
*Tropical Latin America*	433 (379 to 496)	751 (411 to 833)	151.51 (132.39 to 173.50)	0.49 (0.27 to 0.54)	7 (5 to 8)	189 (119 to 633)	2.76 (2.24 to 3.46)	0.09 (0.06 to 0.30)	−98.18 (−98.63 to −97.58)	−81.86 (−89.15 to −38.28)
Brazil	402 (350 to 463)	735 (401 to 813)	146.09 (126.98 to 168.04)	0.49 (0.27 to 0.54)	6 (5 to 7)	186 (117 to 622)	2.46 (2.02 to 3.04)	0.09 (0.06 to 0.30)	−98.31 (−98.73 to −97.77)	−81.66 (−89.01 to −37.99)
Paraguay	31 (20 to 47)	17 (9 to 22)	290.58 (185.68 to 435.72)	0.40 (0.22 to 0.52)	1 (1 to 2)	3 (2 to 11)	9.15 (5.32 to 15.73)	0.04 (0.02 to 0.16)	−96.85 (−98.44 to −93.78)	−89.03 (−94.89 to −44.08)
*Central Latin America*	338 (303 to 381)	459 (427 to 483)	90.24 (80.96 to 101.73)	0.27 (0.25 to 0.29)	9 (7 to 11)	45 (38 to 57)	2.41 (1.91 to 3.06)	0.02 (0.01 to 0.02)	−97.33 (−97.93 to −96.59)	−93.47 (−94.54 to −91.61)
Colombia	81 (65 to 101)	90 (82 to 98)	117.01 (94.78 to 146.31)	0.26 (0.24 to 0.29)	3 (2 to 4)	10 (8 to 13)	5.01 (3.11 to 7.57)	0.02 (0.02 to 0.03)	−95.72 (−97.42 to −93.22)	−92.13 (−93.54 to −89.34)
Costa Rica	0 (0 to 1)	1 (1 to 1)	7.08 (5.56 to 9.00)	0.03 (0.03 to 0.03)	0 (0 to 0)	0 (0 to 0)	0.19 (0.12 to 0.28)	0.00 (0.00 to 0.00)	−97.38 (−98.41 to −95.91)	−95.88 (−96.68 to −93.74)
El Salvador	18 (12 to 25)	31 (11 to 40)	144.26 (101.59 to 201.54)	0.58 (0.21 to 0.75)	0 (0 to 0)	2 (1 to 7)	2.18 (1.14 to 4.12)	0.04 (0.02 to 0.11)	−98.49 (−99.26 to −97.06)	−93.97 (−96.81 to −67.12)
Guatemala	5 (4 to 6)	47 (43 to 53)	17.46 (14.32 to 21.07)	0.52 (0.47 to 0.58)	1 (1 to 1)	5 (4 to 6)	2.55 (1.77 to 3.74)	0.03 (0.02 to 0.04)	−85.39 (−90.54 to −76.86)	−94.59 (−95.99 to −93.04)
Honduras	19 (12 to 30)	16 (8 to 21)	133.39 (81.75 to 208.60)	0.33 (0.16 to 0.43)	2 (1 to 3)	11 (6 to 18)	11.85 (6.02 to 23.44)	0.14 (0.07 to 0.22)	−91.12 (−95.82 to −81.64)	−58.39 (−77.20 to −28.42)
Mexico	200 (177 to 228)	246 (233 to 260)	107.47 (95.24 to 122.50)	0.29 (0.27 to 0.30)	2 (2 to 2)	10 (9 to 13)	1.09 (0.88 to 1.35)	0.01 (0.01 to 0.01)	−98.98 (−99.20 to −98.71)	−97.15 (−97.51 to −96.42)
Nicaragua	9 (7 to 13)	8 (3 to 11)	82.88 (59.56 to 116.46)	0.20 (0.08 to 0.25)	0 (0 to 0)	1 (1 to 1)	0.48 (0.31 to 0.74)	0.01 (0.01 to 0.02)	−99.42 (−99.66 to −99.00)	−93.81 (−95.82 to −77.12)
Panama	2 (1 to 3)	1 (1 to 1)	37.28 (25.89 to 52.26)	0.05 (0.02 to 0.06)	0 (0 to 0)	0 (0 to 1)	0.66 (0.36 to 1.16)	0.01 (0.01 to 0.02)	−98.22 (−99.13 to −96.67)	−73.97 (−81.34 to −39.49)
Venezuela	4 (4 to 5)	19 (17 to 21)	9.88 (8.17 to 11.86)	0.10 (0.09 to 0.10)	1 (1 to 2)	5 (4 to 7)	2.50 (1.70 to 3.83)	0.02 (0.01 to 0.02)	−74.67 (−83.34 to −58.79)	−82.41 (−86.39 to −76.82)
*Southern Sub-Saharan Africa*	31 (23 to 39)	58 (33 to 83)	24.78 (18.04 to 30.95)	0.11 (0.06 to 0.16)	3 (2 to 4)	33 (24 to 60)	2.25 (1.75 to 3.03)	0.04 (0.03 to 0.08)	−90.91 (−93.70 to −85.82)	−60.36 (−76.75 to −24.32)
Botswana	1 (0 to 2)	2 (0 to 7)	18.67 (6.46 to 44.78)	0.13 (0.03 to 0.48)	0 (0 to 0)	1 (0 to 4)	1.78 (0.64 to 3.78)	0.05 (0.01 to 0.18)	−90.48 (−95.61 to −77.14)	−62.33 (−90.32 to 74.78)
Lesotho	3 (1 to 6)	7 (1 to 19)	66.37 (24.80 to 142.61)	0.42 (0.08 to 1.19)	1 (0 to 1)	3 (1 to 8)	11.96 (4.50 to 24.69)	0.14 (0.03 to 0.38)	−81.98 (−92.72 to −56.36)	−65.33 (−86.03 to −10.77)
Namibia	1 (0 to 2)	2 (1 to 5)	24.47 (12.07 to 48.83)	0.17 (0.05 to 0.34)	0 (0 to 0)	1 (0 to 2)	3.07 (1.53 to 5.72)	0.04 (0.02 to 0.08)	−87.47 (−94.64 to −70.09)	−76.65 (−90.27 to −40.09)
South Africa	25 (17 to 33)	39 (22 to 55)	31.04 (20.66 to 40.36)	0.10 (0.06 to 0.15)	2 (1 to 3)	19 (14 to 33)	2.28 (1.75 to 3.01)	0.04 (0.03 to 0.06)	−92.65 (−94.91 to −88.66)	−66.18 (−79.73 to −31.58)
Swaziland	0 (0 to 1)	1 (0 to 3)	14.70 (5.14 to 34.86)	0.13 (0.05 to 0.30)	0 (0 to 0)	1 (0 to 1)	3.38 (1.72 to 6.49)	0.05 (0.02 to 0.10)	−77.03 (−90.25 to −32.14)	−59.99 (−85.78 to 13.85)
Zimbabwe	1 (0 to 2)	7 (3 to 13)	3.18 (1.53 to 6.41)	0.06 (0.03 to 0.12)	0 (0 to 1)	9 (4 to 19)	0.97 (0.44 to 2.01)	0.05 (0.02 to 0.12)	−69.39 (−89.91 to 0.77)	−14.26 (−69.75 to 98.46)
*Southern Latin America*	5 (5 to 6)	39 (35 to 42)	6.72 (5.72 to 8.07)	0.08 (0.07 to 0.09)	1 (0 to 1)	5 (4 to 6)	0.68 (0.51 to 0.94)	0.01 (0.01 to 0.01)	−89.86 (−92.99 to −85.18)	−91.10 (−92.88 to −86.77)
Argentina	4 (4 to 6)	31 (28 to 35)	8.25 (6.84 to 10.24)	0.10 (0.09 to 0.11)	0 (0 to 1)	3 (3 to 5)	0.81 (0.58 to 1.14)	0.01 (0.01 to 0.01)	−90.21 (−93.56 to −85.00)	−91.95 (−93.76 to −87.41)
Chile	1 (1 to 1)	5 (5 to 6)	3.12 (2.55 to 3.96)	0.04 (0.04 to 0.05)	0 (0 to 0)	1 (1 to 1)	0.34 (0.24 to 0.48)	0.01 (0.00 to 0.01)	−89.16 (−93.07 to −83.72)	−86.38 (−88.85 to −80.89)
Uruguay	0 (0 to 0)	2 (1 to 2)	5.57 (4.14 to 7.60)	0.05 (0.05 to 0.06)	0 (0 to 0)	0 (0 to 0)	0.38 (0.25 to 0.57)	0.00 (0.00 to 0.01)	−93.18 (−96.01 to −88.66)	−90.82 (−92.99 to −86.04)
*High-income Asia Pacific*	7 (5 to 11)	53 (49 to 56)	4.77 (3.44 to 7.12)	0.03 (0.03 to 0.03)	0 (0 to 0)	13 (11 to 16)	0.28 (0.22 to 0.36)	0.01 (0.01 to 0.01)	−94.04 (−96.37 to −90.78)	−77.98 (−81.51 to −71.23)
Brunei	0 (0 to 0)	0 (0 to 0)	9.78 (5.43 to 17.15)	0.10 (0.05 to 0.16)	0 (0 to 0)	0 (0 to 1)	1.83 (1.12 to 3.02)	0.06 (0.05 to 0.14)	−81.27 (−91.63 to −57.24)	−36.43 (−58.75 to 17.26)
Japan	1 (1 to 1)	25 (24 to 26)	0.89 (0.81 to 0.96)	0.02 (0.02 to 0.02)	0 (0 to 0)	7 (6 to 9)	0.13 (0.10 to 0.16)	0.01 (0.00 to 0.01)	−85.01 (−88.97 to −81.58)	−71.95 (−75.75 to −64.88)
Singapore	0 (0 to 0)	0 (0 to 0)	1.13 (0.93 to 1.38)	0.01 (0.01 to 0.01)	0 (0 to 0)	0 (0 to 0)	0.12 (0.08 to 0.16)	0.00 (0.00 to 0.00)	−89.75 (−93.82 to −85.35)	−87.21 (−89.37 to −82.43)
South Korea	6 (4 to 10)	27 (24 to 30)	12.44 (8.46 to 19.46)	0.06 (0.06 to 0.07)	0 (0 to 0)	5 (4 to 7)	0.62 (0.45 to 0.84)	0.01 (0.01 to 0.01)	−95.02 (−97.14 to −91.60)	−84.44 (−88.49 to −76.64)
*Central Asia*	2 (2 to 2)	19 (13 to 21)	1.26 (1.11 to 1.42)	0.03 (0.02 to 0.03)	0 (0 to 0)	8 (7 to 12)	0.17 (0.13 to 0.21)	0.01 (0.01 to 0.01)	−86.68 (−89.97 to −82.14)	−64.97 (−70.51 to −40.73)
Armenia	0 (0 to 0)	1 (1 to 1)	0.79 (0.62 to 0.99)	0.03 (0.02 to 0.03)	0 (0 to 0)	0 (0 to 1)	0.09 (0.05 to 0.15)	0.01 (0.01 to 0.02)	−88.02 (−93.31 to −78.56)	−57.24 (−66.91 to −0.22)
Azerbaijan	0 (0 to 0)	2 (1 to 3)	2.05 (1.53 to 2.63)	0.03 (0.02 to 0.04)	0 (0 to 0)	1 (1 to 3)	0.38 (0.24 to 0.60)	0.01 (0.01 to 0.03)	−81.49 (−88.77 to −70.14)	−42.15 (−55.09 to −15.93)
Georgia	0 (0 to 0)	2 (1 to 3)	0.77 (0.59 to 0.99)	0.04 (0.01 to 0.05)	0 (0 to 0)	0 (0 to 1)	0.10 (0.07 to 0.15)	0.01 (0.01 to 0.02)	−86.73 (−91.94 to −78.46)	−71.51 (−79.16 to 11.98)
Kazakhstan	0 (0 to 0)	6 (6 to 7)	0.86 (0.71 to 1.03)	0.04 (0.03 to 0.04)	0 (0 to 0)	0 (0 to 0)	0.11 (0.07 to 0.16)	0.00 (0.00 to 0.00)	−87.25 (−91.75 to −80.97)	−95.29 (−96.24 to −93.19)
Kyrgyzstan	0 (0 to 0)	1 (0 to 1)	0.83 (0.66 to 1.03)	0.02 (0.01 to 0.02)	0 (0 to 0)	1 (1 to 1)	0.15 (0.10 to 0.21)	0.01 (0.01 to 0.01)	−82.44 (−88.80 to −71.24)	−41.86 (−52.76 to 12.24)
Mongolia	0 (0 to 0)	0 (0 to 1)	1.02 (0.54 to 1.69)	0.02 (0.01 to 0.02)	0 (0 to 0)	0 (0 to 0)	0.11 (0.06 to 0.20)	0.01 (0.01 to 0.01)	−88.80 (−94.84 to −74.47)	−35.34 (−53.86 to −1.80)
Tajikistan	0 (0 to 0)	1 (1 to 1)	2.06 (1.54 to 2.71)	0.02 (0.01 to 0.02)	0 (0 to 0)	1 (1 to 1)	0.24 (0.15 to 0.38)	0.01 (0.01 to 0.02)	−88.38 (−93.59 to −78.81)	−47.85 (−61.79 to 14.32)
Turkmenistan	0 (0 to 0)	1 (1 to 1)	3.72 (2.71 to 5.05)	0.02 (0.02 to 0.03)	0 (0 to 0)	1 (1 to 1)	0.19 (0.12 to 0.29)	0.01 (0.01 to 0.02)	−94.94 (−97.08 to −91.33)	−51.31 (−61.06 to −15.08)
Uzbekistan	0 (0 to 1)	4 (3 to 4)	0.77 (0.59 to 0.97)	0.02 (0.01 to 0.02)	0 (0 to 0)	3 (3 to 5)	0.13 (0.08 to 0.20)	0.01 (0.01 to 0.02)	−83.46 (−89.90 to −71.85)	−44.27 (−55.81 to −3.17)
*High-income North America*	3 (3 to 3)	42 (40 to 44)	0.89 (0.82 to 0.96)	0.02 (0.01 to 0.02)	0 (0 to 1)	9 (8 to 11)	0.15 (0.13 to 0.17)	0.00 (0.00 to 0.00)	−83.45 (−86.03 to −80.09)	−82.93 (−85.17 to −78.09)
Canada	0 (0 to 0)	3 (3 to 3)	0.83 (0.71 to 0.98)	0.01 (0.01 to 0.01)	0 (0 to 0)	1 (1 to 1)	0.15 (0.11 to 0.19)	0.00 (0.00 to 0.00)	−82.02 (−87.13 to −75.48)	−80.68 (−84.11 to −74.54)
Greenland	0 (0 to 0)	0 (0 to 0)	1.21 (0.77 to 1.72)	0.02 (0.01 to 0.02)	0 (0 to 0)	0 (0 to 0)	0.13 (0.09 to 0.18)	0.01 (0.01 to 0.01)	−89.52 (−93.98 to −79.44)	−25.14 (−39.86 to −7.14)
United States	3 (3 to 3)	39 (37 to 41)	0.89 (0.83 to 0.97)	0.02 (0.01 to 0.02)	0 (0 to 1)	8 (7 to 10)	0.15 (0.13 to 0.17)	0.00 (0.00 to 0.00)	−83.58 (−86.27 to −80.15)	−83.11 (−85.44 to −78.31)
*Central Europe*	3 (2 to 3)	77 (69 to 86)	1.90 (1.44 to 2.65)	0.06 (0.06 to 0.07)	0 (0 to 0)	11 (10 to 15)	0.14 (0.12 to 0.18)	0.01 (0.01 to 0.01)	−92.49 (−95.05 to −88.86)	−84.68 (−87.32 to −79.88)
Albania	0 (0 to 0)	1 (0 to 1)	3.72 (2.50 to 5.35)	0.03 (0.01 to 0.03)	0 (0 to 0)	0 (0 to 0)	0.16 (0.08 to 0.30)	0.01 (0.01 to 0.01)	−95.67 (−98.10 to −90.79)	−55.84 (−67.08 to −16.27)
Bosnia and Herzegovina	0 (0 to 0)	1 (1 to 2)	0.79 (0.52 to 1.21)	0.03 (0.02 to 0.04)	0 (0 to 0)	0 (0 to 1)	0.10 (0.05 to 0.16)	0.01 (0.01 to 0.02)	−87.27 (−93.50 to −74.63)	−65.64 (−73.32 to −29.18)
Bulgaria	0 (0 to 0)	3 (3 to 4)	0.95 (0.79 to 1.16)	0.04 (0.03 to 0.04)	0 (0 to 0)	0 (0 to 0)	0.13 (0.09 to 0.19)	0.00 (0.00 to 0.01)	−85.85 (−90.89 to −78.47)	−90.99 (−93.06 to −86.45)
Croatia	0 (0 to 0)	6 (5 to 7)	0.87 (0.73 to 1.02)	0.12 (0.10 to 0.15)	0 (0 to 0)	0 (0 to 1)	0.12 (0.09 to 0.16)	0.01 (0.01 to 0.02)	−86.24 (−90.77 to −79.50)	−91.47 (−94.14 to −86.17)
Czech Republic	0 (0 to 0)	2 (1 to 2)	0.90 (0.74 to 1.08)	0.02 (0.01 to 0.02)	0 (0 to 0)	0 (0 to 0)	0.10 (0.07 to 0.14)	0.00 (0.00 to 0.00)	−88.90 (−93.36 to −82.98)	−90.13 (−92.09 to −86.46)
Hungary	0 (0 to 0)	15 (13 to 17)	0.97 (0.82 to 1.13)	0.14 (0.12 to 0.16)	0 (0 to 0)	1 (1 to 1)	0.11 (0.07 to 0.18)	0.01 (0.01 to 0.01)	−88.38 (−93.06 to −81.32)	−94.24 (−95.91 to −90.68)
Macedonia	0 (0 to 0)	0 (0 to 1)	1.28 (0.84 to 1.78)	0.02 (0.01 to 0.03)	0 (0 to 0)	0 (0 to 0)	0.14 (0.08 to 0.23)	0.01 (0.01 to 0.01)	−88.99 (−94.52 to −78.92)	−47.01 (−60.15 to −8.17)
Montenegro	0 (0 to 0)	0 (0 to 0)	0.89 (0.35 to 1.84)	0.01 (0.01 to 0.01)	0 (0 to 0)	0 (0 to 0)	0.09 (0.04 to 0.16)	0.01 (0.01 to 0.01)	−90.34 (−96.85 to −66.82)	−15.73 (−32.03 to −1.04)
Poland	0 (0 to 1)	32 (27 to 37)	1.06 (0.90 to 1.29)	0.08 (0.07 to 0.10)	0 (0 to 0)	4 (3 to 6)	0.10 (0.06 to 0.16)	0.01 (0.01 to 0.01)	−90.52 (−94.40 to −84.39)	−89.14 (−92.54 to −82.51)
Romania	1 (0 to 1)	10 (9 to 10)	2.41 (1.86 to 3.15)	0.04 (0.04 to 0.04)	0 (0 to 0)	2 (2 to 3)	0.25 (0.15 to 0.39)	0.01 (0.01 to 0.02)	−89.79 (−93.77 to −82.85)	−74.15 (−78.72 to −63.71)
Serbia	1 (0 to 2)	7 (2 to 12)	7.26 (2.50 to 16.55)	0.07 (0.02 to 0.13)	0 (0 to 0)	2 (1 to 3)	0.25 (0.16 to 0.40)	0.03 (0.01 to 0.04)	−96.53 (−98.67 to −87.16)	−61.07 (−81.60 to −4.13)
Slovakia	0 (0 to 0)	1 (1 to 1)	0.85 (0.61 to 1.14)	0.01 (0.01 to 0.02)	0 (0 to 0)	1 (1 to 1)	0.11 (0.07 to 0.16)	0.01 (0.01 to 0.01)	−87.52 (−92.85 to −77.49)	−28.85 (−40.42 to −13.91)
Slovenia	0 (0 to 0)	1 (1 to 1)	1.95 (1.56 to 2.40)	0.04 (0.04 to 0.05)	0 (0 to 0)	0 (0 to 0)	0.13 (0.07 to 0.20)	0.00 (0.00 to 0.01)	−93.24 (−96.29 to −89.11)	−92.80 (−95.17 to −87.58)
*Western Europe*	6 (6 to 7)	205 (189 to 223)	1.83 (1.62 to 2.14)	0.05 (0.05 to 0.06)	0 (0 to 1)	36 (28 to 51)	0.14 (0.11 to 0.17)	0.01 (0.01 to 0.01)	−92.39 (−94.28 to −90.27)	−84.72 (−88.33 to −78.23)
Andorra	0 (0 to 0)	0 (0 to 0)	0.49 (0.25 to 0.91)	0.02 (0.01 to 0.03)	0 (0 to 0)	0 (0 to 0)	0.04 (0.02 to 0.07)	0.01 (0.01 to 0.01)	−92.62 (−97.35 to −78.76)	−43.32 (−70.65 to −6.71)
Austria	0 (0 to 0)	1 (1 to 2)	0.88 (0.74 to 1.05)	0.02 (0.02 to 0.02)	0 (0 to 0)	0 (0 to 0)	0.13 (0.09 to 0.17)	0.00 (0.00 to 0.00)	−85.63 (−89.92 to −79.49)	−86.65 (−89.14 to −82.74)
Belgium	0 (0 to 0)	3 (2 to 3)	0.89 (0.74 to 1.08)	0.03 (0.02 to 0.03)	0 (0 to 0)	0 (0 to 0)	0.12 (0.09 to 0.16)	0.00 (0.00 to 0.00)	−86.38 (−90.19 to −80.20)	−90.85 (−92.86 to −87.11)
Cyprus	0 (0 to 0)	0 (0 to 0)	1.99 (1.27 to 3.12)	0.02 (0.01 to 0.03)	0 (0 to 0)	0 (0 to 0)	0.15 (0.10 to 0.22)	0.01 (0.01 to 0.01)	−92.54 (−96.00 to −85.61)	−34.85 (−54.76 to −12.66)
Denmark	0 (0 to 0)	1 (1 to 1)	0.93 (0.78 to 1.12)	0.01 (0.01 to 0.02)	0 (0 to 0)	0 (0 to 0)	0.14 (0.09 to 0.19)	0.00 (0.00 to 0.00)	−85.50 (−90.64 to −79.03)	−84.74 (−87.69 to −78.99)
Finland	0 (0 to 0)	1 (1 to 1)	0.92 (0.76 to 1.11)	0.01 (0.01 to 0.02)	0 (0 to 0)	0 (0 to 0)	0.12 (0.07 to 0.16)	0.00 (0.00 to 0.00)	−87.48 (−92.58 to −80.86)	−80.19 (−85.30 to −71.17)
France	1 (0 to 1)	60 (48 to 74)	0.90 (0.76 to 1.05)	0.11 (0.08 to 0.13)	0 (0 to 0)	5 (3 to 9)	0.11 (0.07 to 0.17)	0.01 (0.01 to 0.01)	−87.71 (−92.61 to −80.46)	−92.37 (−95.48 to −85.52)
Germany	1 (0 to 1)	12 (11 to 14)	0.87 (0.74 to 1.03)	0.02 (0.01 to 0.02)	0 (0 to 0)	2 (2 to 3)	0.12 (0.08 to 0.17)	0.00 (0.00 to 0.00)	−86.58 (−90.89 to −80.15)	−84.73 (−88.04 to −79.83)
Greece	0 (0 to 0)	3 (3 to 3)	1.76 (1.39 to 2.29)	0.03 (0.03 to 0.03)	0 (0 to 0)	2 (2 to 3)	0.45 (0.30 to 0.70)	0.02 (0.01 to 0.03)	−74.35 (−84.05 to −59.00)	−36.33 (−50.32 to −5.94)
Iceland	0 (0 to 0)	0 (0 to 0)	0.81 (0.66 to 0.97)	0.01 (0.01 to 0.01)	0 (0 to 0)	0 (0 to 0)	0.09 (0.06 to 0.13)	0.00 (0.00 to 0.00)	−88.30 (−92.88 to −82.19)	−83.00 (−86.73 to −73.58)
Ireland	0 (0 to 0)	0 (0 to 0)	0.90 (0.74 to 1.08)	0.01 (0.01 to 0.01)	0 (0 to 0)	0 (0 to 0)	0.10 (0.07 to 0.13)	0.00 (0.00 to 0.00)	−89.16 (−92.48 to −85.01)	−90.46 (−92.19 to −87.34)
Israel	0 (0 to 0)	0 (0 to 0)	0.91 (0.76 to 1.10)	0.01 (0.01 to 0.01)	0 (0 to 0)	0 (0 to 0)	0.11 (0.08 to 0.15)	0.00 (0.00 to 0.00)	−87.79 (−91.37 to −82.57)	−87.95 (−89.80 to −84.54)
Italy	0 (0 to 1)	60 (52 to 70)	1.15 (0.95 to 1.39)	0.11 (0.09 to 0.12)	0 (0 to 0)	20 (14 to 30)	0.13 (0.09 to 0.20)	0.03 (0.02 to 0.05)	−88.41 (−92.76 to −82.11)	−70.55 (−80.46 to −54.07)
Luxembourg	0 (0 to 0)	0 (0 to 0)	1.62 (1.33 to 2.02)	0.04 (0.04 to 0.05)	0 (0 to 0)	0 (0 to 0)	0.12 (0.08 to 0.17)	0.00 (0.00 to 0.01)	−92.45 (−95.10 to −88.89)	−89.60 (−92.56 to −84.56)
Malta	0 (0 to 0)	1 (0 to 1)	6.80 (5.16 to 9.05)	0.15 (0.13 to 0.17)	0 (0 to 0)	0 (0 to 0)	0.40 (0.29 to 0.56)	0.01 (0.01 to 0.01)	−94.12 (−96.09 to −91.20)	−93.06 (−94.47 to −90.83)
Netherlands	0 (0 to 0)	2 (2 to 2)	0.90 (0.75 to 1.08)	0.01 (0.01 to 0.01)	0 (0 to 0)	0 (0 to 0)	0.13 (0.10 to 0.18)	0.00 (0.00 to 0.00)	−85.45 (−90.09 to −78.96)	−83.47 (−86.22 to −78.11)
Northern Ireland	0 (0 to 0)	0 (0 to 0)	0.88 (0.73 to 1.04)	0.02 (0.01 to 0.02)	0 (0 to 0)	0 (0 to 0)	0.16 (0.12 to 0.23)	0.00 (0.00 to 0.00)	−81.37 (−87.23 to −72.75)	−85.75 (−88.33 to −81.58)
Norway	0 (0 to 0)	1 (0 to 1)	0.93 (0.78 to 1.09)	0.01 (0.01 to 0.01)	0 (0 to 0)	0 (0 to 0)	0.11 (0.07 to 0.15)	0.00 (0.00 to 0.00)	−87.82 (−92.00 to −83.13)	−83.90 (−87.29 to −78.40)
Portugal	2 (1 to 2)	17 (15 to 20)	19.52 (15.07 to 26.03)	0.17 (0.15 to 0.20)	0 (0 to 0)	1 (0 to 1)	0.24 (0.18 to 0.32)	0.01 (0.00 to 0.01)	−98.76 (−99.20 to −98.15)	−96.40 (−97.47 to −94.32)
Scotland	0 (0 to 0)	1 (1 to 1)	0.85 (0.72 to 1.00)	0.01 (0.01 to 0.02)	0 (0 to 0)	0 (0 to 0)	0.13 (0.09 to 0.18)	0.00 (0.00 to 0.00)	−85.24 (−90.09 to −78.92)	−84.82 (−87.47 to −80.10)
Spain	2 (1 to 2)	32 (28 to 36)	5.37 (4.15 to 7.08)	0.08 (0.07 to 0.09)	0 (0 to 0)	3 (2 to 4)	0.19 (0.14 to 0.26)	0.01 (0.00 to 0.01)	−96.49 (−97.75 to −94.74)	−93.33 (−95.05 to −89.61)
Sweden	0 (0 to 0)	1 (1 to 2)	1.23 (1.03 to 1.43)	0.02 (0.02 to 0.02)	0 (0 to 0)	0 (0 to 0)	0.14 (0.09 to 0.19)	0.00 (0.00 to 0.00)	−88.49 (−92.60 to −83.72)	−87.91 (−90.32 to −82.15)
Switzerland	0 (0 to 0)	1 (1 to 1)	0.92 (0.77 to 1.13)	0.01 (0.01 to 0.01)	0 (0 to 0)	0 (0 to 1)	0.14 (0.10 to 0.19)	0.00 (0.00 to 0.01)	−85.24 (−90.28 to −78.75)	−68.16 (−78.27 to −51.56)
United Kingdom	1 (0 to 1)	8 (7 to 8)	0.84 (0.77 to 0.92)	0.01 (0.01 to 0.01)	0 (0 to 0)	1 (1 to 1)	0.14 (0.11 to 0.16)	0.00 (0.00 to 0.00)	−83.86 (−86.93 to −80.33)	−86.47 (−88.07 to −83.45)
*Australasia*	0 (0 to 0)	3 (3 to 3)	0.89 (0.77 to 1.02)	0.01 (0.01 to 0.02)	0 (0 to 0)	1 (1 to 1)	0.12 (0.09 to 0.15)	0.00 (0.00 to 0.00)	−86.79 (−90.17 to −82.09)	−84.68 (−87.76 to −78.32)
Australia	0 (0 to 0)	2 (2 to 3)	0.89 (0.75 to 1.05)	0.01 (0.01 to 0.02)	0 (0 to 0)	1 (0 to 1)	0.11 (0.09 to 0.15)	0.00 (0.00 to 0.00)	−87.17 (−90.72 to −81.94)	−84.10 (−87.58 to −76.88)
New Zealand	0 (0 to 0)	1 (0 to 1)	0.88 (0.74 to 1.04)	0.01 (0.01 to 0.02)	0 (0 to 0)	0 (0 to 0)	0.13 (0.10 to 0.18)	0.00 (0.00 to 0.00)	−84.79 (−89.05 to −78.92)	−87.55 (−89.66 to −83.85)
*Eastern Europe*	2 (2 to 2)	92 (82 to 104)	0.80 (0.71 to 0.91)	0.04 (0.04 to 0.05)	0 (0 to 0)	10 (8 to 18)	0.11 (0.09 to 0.14)	0.00 (0.00 to 0.01)	−85.89 (−89.34 to −81.56)	−88.35 (−90.91 to −81.16)
Belarus	0 (0 to 0)	4 (1 to 4)	0.82 (0.60 to 1.11)	0.04 (0.01 to 0.04)	0 (0 to 0)	1 (1 to 2)	0.06 (0.03 to 0.10)	0.01 (0.01 to 0.02)	−92.79 (−96.35 to −86.33)	−67.06 (−75.95 to 4.65)
Estonia	0 (0 to 0)	0 (0 to 0)	0.97 (0.76 to 1.20)	0.02 (0.02 to 0.02)	0 (0 to 0)	0 (0 to 0)	0.08 (0.04 to 0.12)	0.00 (0.00 to 0.00)	−91.91 (−95.35 to −87.28)	−92.63 (−94.40 to −89.83)
Latvia	0 (0 to 0)	0 (0 to 1)	0.88 (0.72 to 1.05)	0.02 (0.02 to 0.02)	0 (0 to 0)	0 (0 to 0)	0.08 (0.05 to 0.13)	0.00 (0.00 to 0.00)	−90.42 (−94.96 to −84.07)	−91.24 (−93.16 to −87.12)
Lithuania	0 (0 to 0)	1 (1 to 2)	0.83 (0.68 to 0.99)	0.04 (0.04 to 0.05)	0 (0 to 0)	0 (0 to 0)	0.09 (0.05 to 0.13)	0.01 (0.01 to 0.01)	−89.39 (−93.98 to −84.39)	−81.23 (−86.05 to −71.74)
Moldova	0 (0 to 0)	1 (1 to 1)	0.77 (0.61 to 0.96)	0.01 (0.01 to 0.02)	0 (0 to 0)	0 (0 to 0)	0.08 (0.05 to 0.13)	0.00 (0.00 to 0.00)	−89.39 (−94.00 to −81.79)	−92.61 (−94.03 to −88.46)
Russia	1 (1 to 1)	33 (29 to 39)	0.79 (0.67 to 0.93)	0.02 (0.02 to 0.03)	0 (0 to 0)	4 (4 to 6)	0.12 (0.09 to 0.15)	0.00 (0.00 to 0.00)	−84.95 (−88.80 to −79.41)	−86.72 (−89.17 to −83.18)
Ukraine	0 (0 to 1)	52 (45 to 61)	0.82 (0.63 to 1.04)	0.10 (0.09 to 0.12)	0 (0 to 0)	4 (3 to 10)	0.11 (0.06 to 0.19)	0.01 (0.01 to 0.02)	−86.63 (−93.10 to −77.12)	−90.48 (−93.92 to −78.95)

Between 1990 and 2015, the global mortality rate due to neonatal tetanus dropped by 90% and that due to non-neonatal tetanus dropped by 81% (Table 1). At the country level, the decline in neonatal tetanus mortality rate varied from −47% in Somalia to −95% in Angola in sub-Saharan Africa. The decline in tetanus mortality rate after the neonatal period varied from −0.12% in South Sudan to −92% in Mauritania in sub-Saharan Africa (Table 1).

**Fig. 2:**
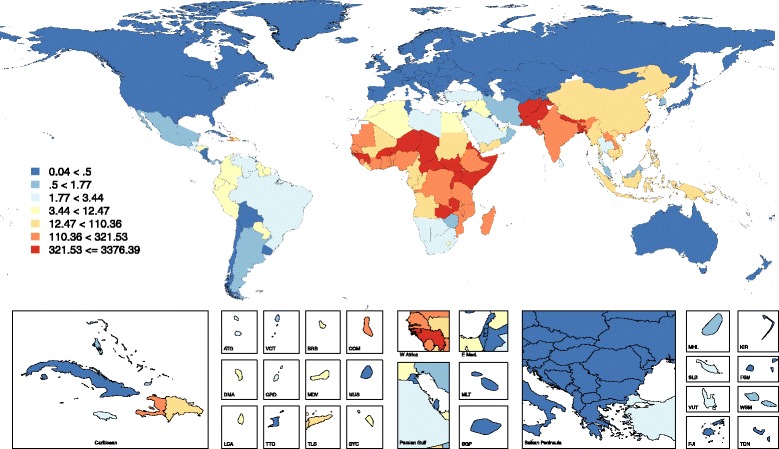
Neonatal tetanus mortality rate (per 100, 000 population), both sexes, 2015

**Fig. 3:**
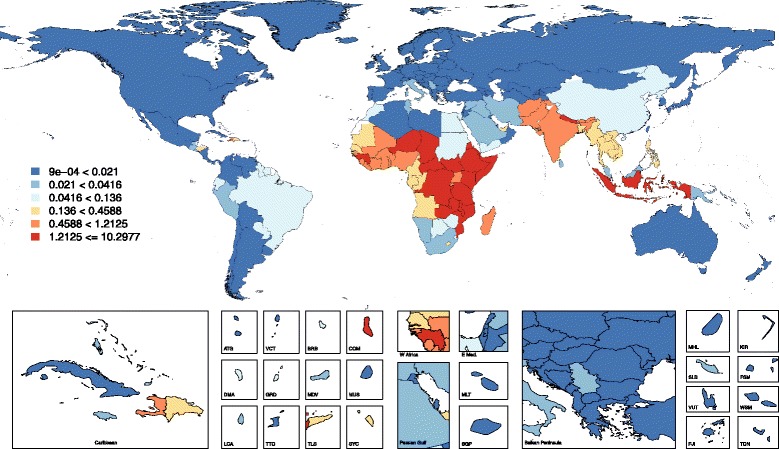
Non-neonatal tetanus mortality rate (per 100, 000 population), both sexes, 2015

There were also substantial between-country variations in tetanus mortality rates (Figs. 2 and 3). For example, neonatal tetanus mortality rates per 100,000 people varied from 3,376.4 (1,731.6 to 6,447.9) in Somalia to 1.0 (0.4 to 2.0) in Zimbabwe in sub-Saharan Africa in 2015 (Table 1). Tetanus mortality per 100,000 people after the neonatal period varied from 10.3 (3.6 to 23.7) in Somalia to 0.04 (0.03 to 0.06) in South Africa in the same year (Table 1).

Although both neonatal and non-neonatal tetanus deaths were concentrated in low and middle countries, a small number of deaths from non-neonatal tetanus continued to occur in high-income countries. We estimated 36 (UI: 28 to 51) deaths in Western Europe, 13 (UI: 11–16) deaths in high-income Asia Pacific, and 9 (UI: 8 to 11) deaths in high-income North America due to tetanus in 2015 (Table 1): most of these deaths occurred in adults, especially among elderly people. More detailed results showing the location-year-age-sex specific distributions of tetanus mortality from 1990 to 2015 in 5-year interval are viewable in an interactive online visualization tool at http://vizhub.healthdata.org/gbd-compare.

## Discussion

Exceptional progress has been made over the past two decades in reducing mortality from tetanus worldwide. Nevertheless, mortality from tetanus was still unnecessarily high in a number of low and middle income countries in 2015. The scale-up of immunization coverage to prevent maternal and neonatal tetanus represents a huge success of a collective effort. However, the scale-up has not been universal, with low vaccination coverage being documented in several countries [6, 12, 13]. Constraints related to financial and human resources and difficulty vaccinating people in hard-to-reach rural areas were among the factors influencing the tetanus toxoid vaccine coverage [12].

Tetanus mortality rates were the highest among neonates in low and middle income countries, indicating failures of health systems to provide immunization, antenatal care, and clean deliveries for all births. Mortality rates from tetanus after the neonatal period were much higher in low and middle income countries compared with high income countries, but a small number of deaths continued to occur in high income countries due to low vaccination coverage in adults [14, 15]. Our findings showed that age-standardized mortality from tetanus was higher among males than females globally. Previous studies have also reported male sex as a risk factor for both neonatal and non-neonatal tetanus [16, 17]. Although the exact reason is not clear, possible explanation for the increased risk of tetanus among newborn boys include medical-care seeking for boys, differential cord care, maternal recall, and circumcision practices [13, 16]. Among adults, occupational exposure and relatively lower vaccination coverage in men were among the reasons for the increased risk [17].

A main limitation of this study concerns the poor availability of data in many sub-Saharan African countries where tetanus mortality is most common. For countries without reliable vital registration systems, our analysis relies on verbal autopsy data. Variations in analytical methods and the instrument used for collection of verbal autopsy data may also introduce measurement bias and reduce the comparability of tetanus cause-of-death data across countries. Estimating tetanus mortality for every geography over time is challenging especially for those with sparse or no data. We applied sophisticated modeling methods, borrowing strength across geography and covariates to help predict for locations and years with limited data. Accordingly, the estimates for a geography with sparse data are reflected by wider uncertainty intervals (Detailed information on data availability, model estimates and uncertainty intervals for each region and country are available online at http://vizhub.healthdata.org/cod/). New data for countries, especially in the sub-Saharan African region would narrow the uncertainty in the tetanus mortality estimates for countries in the region.

## Conclusions

Up-to-date information on the levels and trends of tetanus mortality is critical to guide prevention and intervention efforts. Despite the availability of a safe, inexpensive, and effective vaccine, our findings on tetanus mortality suggest that the vaccine is not fully utilized. Despite the general decline in tetanus mortality, tens of thousands of lives could still be saved by scaling up interventions.

## Additional file

Additional file 1: Tetanus data sources and citations. (XLS 114 kb)
